# Supplementary material for: Splice-Junction-Based Mapping of Alternative Isoforms in the Human Proteome
Source: Cell Rep. Author manuscript; Available in PMC 2020 Jan 15. (PMC6961840; doi:10.1016/j.celrep.2019.11.026)
Supplement: 2 [file NIHMS1546469-supplement-2.zip › DF1/PXD010154/Heart/Heart_38_NEXN_QEMGEEDDVDVRPAR.pdf]

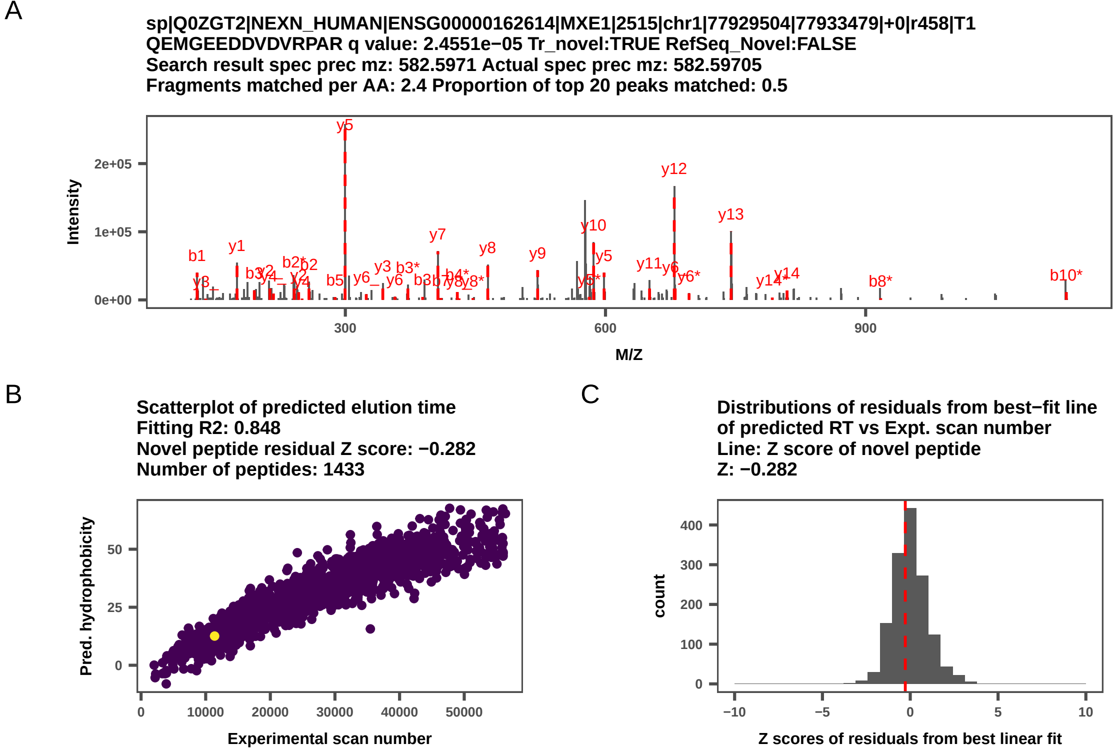
A
sp|Q0ZGT2|NEXN_HUMAN|ENSG00000162614|MXE1|2515|chr1|77929504|77933479|+0|r458|T1
QEMGEEDDVDVRPAR q value: 2.4551e−05 Tr_novel:TRUE RefSeq_Novel:FALSE
Search result spec prec mz: 582.5971 Actual spec prec mz: 582.59705
Fragments matched per AA: 2.4 Proportion of top 20 peaks matched: 0.5
Intensity
2e+05
1e+05
0e+00
M/Z
300
600
900
y5
y12
y13
y10
y7
y8
y9
y1
b1
y11
y14
b8*
b10*
B
Scatterplot of predicted elution time
Fitting R2: 0.848
Novel peptide residual Z score: −0.282
Number of peptides: 1433
Pred. hydrophobicity
Experimental scan number
0
25
50
10000
20000
30000
40000
50000
C
Distributions of residuals from best−fit line
of predicted RT vs Expt. scan number
Line: Z score of novel peptide
Z: −0.282
count
Z scores of residuals from best linear fit
100
200
300
400
−10
−5
5
10
